# Establishment of transgene-free induced pluripotent stem cells reprogrammed from human stem cells of apical papilla for neural differentiation

**DOI:** 10.1186/scrt134

**Published:** 2012-10-24

**Authors:** Xiao-Ying Zou, Hsiao-Ying Yang, Zongdong Yu, Xiao-Bing Tan, Xing Yan, George T-J Huang

**Affiliations:** 1 Department of Endodontics, Boston University Henry M. Goldman School of Dental Medicine, Boston, MA 02118, USA; 2 Department of Cariology, Endodontology and Operative Dentistry, School and Hospital of Stomatology, Peking University, Beijing, 100081, P. R. China; 3 Department of Bioscience Research, College of Dentistry, University of Tennessee Health Science Center. Memphis, TN, USA; 4 Department of Stomatology, 1st People's Hospital of Yunnan Province, Kunming, Yunnan, 650032, P. R. China; 5 Department of Stomatology, Beijing Friendship Hospital (Second Clinical School), Capital Medical University, Beijing 100050, P.R. China

## Abstract

**Introduction:**

Induced pluripotent stem cells (iPSCs) are a potent cell source for neurogenesis. Previously we have generated iPSCs from human dental stem cells carrying transgene vectors. These exogenous transgenes may affect iPSC behaviors and limit their clinical applications. The purpose of this study was to establish transgene-free iPSCs (TF-iPSCs) reprogrammed from human stem cells of apical papilla (SCAP) and determine their neurogenic potential.

**Methods:**

A single lentiviral 'stem cell cassette' flanked by the loxP site (hSTEMCCA-loxP), encoding four human reprogramming factors, *OCT4, SOX2, KLF4*, and *c-MYC*, was used to reprogram human SCAP into iPSCs. Generated iPSCs were transfected with plasmid pHAGE2-EF1α-Cre-IRES-PuroR and selected with puromycin for the TF-iPSC subclones. PCR was performed to confirm the excision of hSTEMCCA. TF-iPSC clones did not resist to puromycin treatment indicating no pHAGE2-EF1α-Cre-IRES-PuroR integration into the genome. *In vitro *and *in vivo *analyses of their pluripotency were performed. Embryoid body-mediated neural differentiation was undertaken to verify their neurogenic potential.

**Results:**

TF-SCAP iPSCs were generated *via *a hSTEMCCA-loxP/Cre system. PCR of genomic DNA confirmed transgene excision and puromycin treatment verified the lack of pHAGE2-EF1α-Cre-IRES-PuroR integration. Transplantation of the TF-iPSCs into immunodeficient mice gave rise to teratomas containing tissues representing the three germ layers -- ectoderm (neural rosettes), mesoderm (cartilage and bone tissues) and endoderm (glandular epithelial tissues). Embryonic stem cell-associated markers TRA-1-60, TRA-2-49 and OCT4 remained positive after transgene excision. After neurogenic differentiation, cells showed neural-like morphology expressing neural markers *nestin, βIII-tubulin, NFM, NSE, NeuN, GRM1, NR1 *and *CNPase*.

**Conclusions:**

TF-SCAP iPSCs reprogrammed from SCAP can be generated and they may be a good cell source for neurogenesis.

## Introduction

Stem cells from apical papilla (SCAP) are derived from the developing tissue at the apex of a tooth root termed apical papilla [1,2]. These cells exhibit mesenchymal stem cell (MSC) properties with multi-lineage differentiation potential and they express several neural markers when grown in neurogenic cell culture, including βIII-tubulin, glutamic acid decarboxylase (GAD), NeuN, nestin, neurofilament medium chain (NFM), neuron-specific enolase (NSE), and 2', 3'-cyclic nucleotide-3'-phosphodiesterase (CNPase) [2,3]. SCAP are considered a type of cell source for odontoblasts responsible for root development and they are capable of regenerating pulp and dentin tissues *in vivo *[4]. SCAP are highly robust in terms of relatively high population doubling and telomerase activities [1,2]. We have previously shown that three types of dental stem cells, SCAP, DPSCs (dental pulp stem cells) and SHED (stem cells from human exfoliated deciduous teeth) can be easily reprogrammed into induced pluripotent stem cells (iPSCs) at a higher reprogramming rate than dermal fibroblasts using Thomson's four factors, *LIN28, NANOG, OCT4 *and *SOX2 *and their vector system [5].

iPSCs have tremendous medical applications and are very similar to embryonic stem cells (ESCs) [6,7]. However, it was realized that iPSCs generated by viral vector transduction either using Yamanaka's four factors, *c-MYC, KLF4, OCT4, SOX2 *[8,9] or Thomson's four factors prevent iPSCs from being more similar to ESCs because the transgene-carrying iPSCs have a different profile at global gene expression and epigenetic levels, and have altered differentiation into functional cell types [10-12]. Furthermore, it is obvious that carrying oncogenes such as *c-MYC *raises a safety concern for their clinical use [13-16].

Tremendous efforts have been made to deliver the reprogramming factors without viral vector integration. The approaches include transient expression using adenoviral or nonviral vectors [17,18], removing the integrated vectors using piggyBac transposition [19,20], minicircle DNA [21], and non-integrating episomal vectors [15,22,23]. However, the reprogramming efficiencies both in human or mouse systems were very low ranging from 0.00005% to 0.039% and most cases were at the lower end. The approach of non-integrating episomal vectors reported by Yu *et al. *[22] required three individual plasmids carrying a total of seven factors, including the oncogene *SV40*, and has not been shown to reprogram cells successfully from adult donors. Using recombinant protein-based four factors [24,25], synthesized mRNA [26], and Sendai virus [27] to generate iPSCs has also been reported. Unfortunately, the protein transduction method is extremely difficult, labor-intensive and time-consuming at present, and modifying Sendai virus vectors or preparing synthesized RNA is technically demanding.

Recently, a single lentiviral 'stem cell cassette' (STEMCCA) carrying all four Yamanaka's factors was developed for the efficient generation of iPSCs from mouse postnatal fibroblasts [28]. Subsequently, the STEMCCA was modified into an excisable single polycistronic vector containing loxP sites. Most importantly, the excision of the vector system after reprogramming improved the iPSC differentiation potential [29]. The STEMCCA-loxP was then humanized to carry four human reprogramming factors, *OCT4, SOX2, KLF4*, and *c-MYC *(designated as hSTEMCCA-loxP) and has been shown to reprogram human fibroblasts successfully [30]. This excisable single polycistronic vector system provides a high efficiency in reprogramming fibroblasts into iPSCs (mouse: 0.5%; human: 0.1% to 1.5%). To utilize iPSCs for clinical applications or to understand the biology of these cells, removal of exogenously introduced genes in these cells is a critical step, given the premise that iPSCs are independent of exogenous reprogramming transgene expression and retain their pluripotency following factor withdrawal. Using the single vector STEMCCA and Cre/loxP system, transgene-free (TF) iPSCs can be generated which possess pluripotent characteristics [29,30]. In the present study, we applied this system and technology to generate TF iPSCs from SCAP and examined their neurogenic potential as the first step toward generating and characterizing TF iPSCs from other oral cells/stem cells.

## Methods

### Cell culture

Human SCAP primary cultures were established as previously described [2]. In brief, freshly extracted teeth were collected from healthy donors (16- to 24-years old) in the Oral Surgery Clinics at Boston University based on an exempt protocol approved by the Boston University Medical Institutional Review Board (#H-28882). Apical papilla from immature permanent teeth were minced and digested in a solution of 3 mg/mL collagenase type I and 4 mg/mL dispase (Sigma-Aldrich, St. Louis, MO, USA) for 30 to 60 minutes at 37°C. The digested mixtures were passed through a 70-μm cell strainer (Falcon, BD Labware, Franklin Lakes, NJ, USA) to obtain single cell suspensions. Cells were seeded into six-well plates and cultured with α-minimum essential medium (α-MEM; Life Technologies/Invitrogen, Grand Island, NY, USA) supplemented with 15% fetal bovine serum (FBS; Gemini Bio-Products, Inc., Woodland, CA, USA), 2 mM L-glutamine, 100 μM L-ascorbic acid-2-phosphate, 100 U/mL penicillin-G, 100 mg/mL streptomycin, and 0.25 mg/mL fungizone (Gemini Bio-Products, Inc., West Sacramento, CA, USA) and maintained in 5% CO_2 _at 37°C. Colony formation units of fibroblastic cells (CFU-F) were normally observed within 1 to 2 weeks after cell seeding and were passaged at a 1:3 ratio when they reached approximately 70% confluence. Heterogeneous populations of SCAP were frozen and stored in liquid nitrogen at passages 0 to 2 (p0 to p2). Cells were thawed and expanded for experimentation. These heterogeneous populations of adherent, clonogenic dental stem/progenitor cells were routinely tested for their cell surface marker expression with flow cytometry and they were positive for STRO-1, CD146, CD73, CD90, and CD105 and negative for CD14, CD34, and CD45, which are typical features of MSCs [3]. Mouse embryonic fibroblasts (MEFs) were used as the feeder cells isolated from E13.5 embryos of CF1 pregnant mice according to the NIH standard protocol [31] and cultured in (D)MEM supplemented with 10% FBS. All animal procedures followed a protocol approved by the Institutional Animal Care and Use Committee (IACUC) at Boston University (protocol# AN15026).

### Transduction and reprogramming of SCAP

Lentiviruses were produced in 293T packaging cells with five plasmid cotransfection, concentrated by ultracentrifugation and titered as previously described [29,30]. Viral titers of approximately 1 × 10^8 ^TU (transducing unit)/ml were employed for reprogramming experiments. Heterogeneous primary human SCAP at p3 were transduced with lentiviral vectors hSTEMCCA-loxP (a polycistronic single vector carrying all four human reprogramming factors, *c-MYC, KLF4, OCT4 and SOX2*) in the presence of polybrene (5 μg/mL). Within six days, 4 × 10^4 ^transduced SCAP were trypsinized and seeded onto mitomycin C-inactivated MEFs on a 10-cm gelatin-coated culture dish. The next day, the medium was changed to human embryonic stem cell (hESC) medium (80% (D)MEM/F12, 20% knock-out serum replacement, 1x non-essential amino acid, 1 mM L-glutamine, 0.1 μM β-mercaptoethanol) containing 4 ng/mL basic fibroblast growth factor (bFGF). Within two weeks, a few cell colonies resembling ESC colonies began to emerge (they were considered as p0 prospective iPSCs). Emerged colonies were manually isolated/passaged 28 days post-viral vector transduction and expanded on MEF feeders in hESC medium. Each colony was manually subcloned and grown/expanded separately into wells of 12-well plates. The expanded SCAP iPSCs at p2 were used for further Cre-mediated excision of hSTEMCCA.

### Excision of hSTEMCCA

The transfection of SCAP-iPSC colonies for the excision of the transgene/vector was performed using the Hela Monster transfection reagent (Mirus, Madison, WI, USA) according to the manufacturer's instructions. Briefly, 60% to 70% confluent p2 SCAP iPSCs were cultured on puromycin resistant MEFs (DR4 MEF, GlobalStem, Rockville, MD, USA) in six-well plates. The cells were then exposed to 2.5 mL/well hESC medium containing 4 ng/mL bFGF, 2.5 μg pHAGE2-Cre-IRES-PuroR plasmid DNA, 7.5 μl Trans IT Hela reagent and 5 μl MONSTER reagent. After 24 hours, the medium was changed to hESC medium and the cultures incubated for about an additional 6 hours, after which selection of the transfected SCAP iPSCs with puromycin (1.2 μg/mL) began and lasted for 48 hours. The medium containing puromycin was changed every 24 hours. Fresh hESC medium was used after puromycin treatment and changed daily. New SCAP iPSC colonies re-emerged in two to four days. On days 11 to 14, five newly emerged colonies from each well were picked and transferred into new MEFs plates and grown/expanded individually. Genomic DNA from each subclone was extracted and PCR performed to verify for the excision of the hSTEMCCA. The primers used and the PCR conditions are as follows: *c-MYC *(forward primer): 5' -GGA ACT CTT GTG CGT AAG TCG ATA G-3'; *WPRE *(reverse primer) 5'-GGA GGC GGC CCA AAG GGA GGA GAT CCG-3'; 95°C for 3 minutes; followed by 33 cycles of 94°C for 30 seconds, 60°C for 30 seconds and 72°C for 5 minutes. The PCR products were examined by electrophoresis on an agarose gel. Verified transgene free clones were named TF-SCAP iPSCs.

To verify that there was no integration of pHAGE2-Cre-IRES-PuroR plasmid DNA into the genome of TF-SCAP iPSCs, these cells were grown on DR4 MEFs in the presence of puromycin (1.2 μg/mL). Total cell death indicated the lack of plasmid integration.

### Immunocytofluorescence

iPSC colonies grown on MEFs in 12-well plates were fixed in 100% ice cold methanol, incubated in blocking buffer (32.5 mM NaCl, 3.3 mM Na_2_HPO_4_, 0.76 mM KH_2_PO_4_, 1.9 mM NaN3, 0.1% (w/v) BSA, 0.2% (v/v) Triton X-100, 0.05% (v/v) Tween 20, and 5% goat serum) for 30 minutes followed by the addition of the following antibodies for 1 hour at room temperature: mouse anti-human TRA-1-60, TRA-2-49 (both from Chemicon/Millipore, Temecula, CA, USA) and OCT4 (Santa Cruz Biotech., Santa Cruz, CA, USA). After washing, cultures were incubated with anti-mouse antibodies Alexa Fluor 594 (Invitrogen) for 1 hour at room temperature and the cell nuclei stained with 4',6-diamidino-2-phenylindole (DAPI, Invitrogen). Images were analyzed under a fluorescence microscope.

### Teratomas formation

SCAP iPSCs and TF-SCAP iPSCs at approximately 70% confluence in a six-well plate were harvested by collagenase IV treatment, collected into tubes, centrifuged, and the pellets resuspended in the (D)MEM/F12 and Matrigel (3:1) solution. The resuspended cells were injected intramuscularly into the right and/or left hind legs of a SCID mouse (SCID, NOD.CB17-Prkdc-scid/J; Jackson Laboratory, Bar Harbor, ME, USA). Seven to nine weeks after injection, tumors were resected, fixed with PBS containing 4% paraformaldehyde and processed. Paraffin-embedded tissue was sectioned and stained with H & E for histologic analysis.

### Neurogenic differentiation *in vitro *of TF- SCAP iPSCs

TF-SCAP iPSC colonies were grown on Matrigel with TESR2 medium (Stemcell Technology, Vancouver, BC, Canada). Subsequently, iPSC colonies were treated with dispase (1 mg/mL) for seven minutes, resuspended in (D)MEM/F12 supplemented with 20% FBS and 2 mM glutamine. The colonies were collected and plated into Ultra Low six-well plates to form embryoid bodies (EBs) for four days. EBs were collected and plated into Matrigel-coated wells of six-well plates. After cell attachment, the medium was changed to neurogenic differentiation medium ((D)MEM supplemented with B27 and 20 ng/mL bFGF). The cells were incubated in the differentiation medium for eight days and subsequently stained for the neural markers βIII-tubulin (TUJ1; Sigma-Aldrich), nestin (Millipore) and neurofilament medium chain (NFM; Invitrogen). Cells were fixed in 100% ice cold methanol for 20 minutes and washed with PBS 3 times. Triton X-100 (0.1%) was applied to permeabilize the cells for 15 minutes followed by washing with PBS 3 times. Fixed cells were blocked with 5% goat serum for 1 hour. Primary mouse monoclonal antibodies to human βIII-tubulin, nestin and NFM (all at1:200) were applied and incubated at 4°C overnight. After washing with PBS 3 times, secondary goat anti-mouse Alexa Fluor 594 antibodies (Invitrogen) were added and incubated at room temperature for 1 hour, and cell nuclei were stained with DAPI (1:10,000) (Invitrogen) [5]. Staining was examined under a fluorescence microscope. RT-PCR analysis of neurogenic gene expression was performed. Total RNA was isolated using RNeasy Mini Kit (Qiagen, Vaenicia, CA, USA) from TF-SCAP iPSCs grown on Matrigel with TESR2 medium and neural-like cells after neurogenesis. cDNA was synthesized with a SuperScript III RT-PCR kit (Invitrogen) followed by PCR using Taq DNA Polymerase (Invitrogen) to detect the human neural gene expression listed in Table 1.

**Table 1:** Primers for RT-PCR.

Gene	GenBank Accession	Primer (5'--- 3')	Product size (bp)
		Forward	
		Reverse	
*Nestin*	NM_006617	AAC AGC GAC GGA GGT CTC TA TTC TCT TGT CCC GCA GAC TT	220
*βIII-tubulin*	NM_001197181	CAG ATG TTC GAT GCC AAG AA GGG ATC CAC TCC ACG AAG TA	164
*NFM*	NM_005382	CGA CCT CAG CAG CTA CCA GGA CAC CAG TGA TGC TTC CTG CAA ATG TGC T	200
*NSE*	NM_001975	GTC CCA CGT GTC TTC CAC TT TGG GAT CTA CAG CCA CAT GA	236
*NeuN*	NM_001082575	CTT ACG GAG CGG TCG TGT AT AGA AGG AAA CGG TGG AAG GT	215
*GRM1*	NM_000838	TGA GGG TTG TCC CTT CTG AC GGA AGC CTC TCT CGG AGT TT	241
*NR1-1*	NM_000832	AAG CCT CGA GGG TAC CAG AT AGC TTG ATG AGC AGG TCG AT	236
*CNPase*	NM_033133	GTG GAG CAC AAA AGC CTC TC AAG TTT CCC ATG TGG CTG AC	232
*GFAP*	NM_001131019	GCA GAT TCG AGG GGG CAA AA GAA CTC AGG GGG ATT GGG AG	221
*GAPDH*	NM_002046	CAA GGC TGA GAA CGG GAA GC AGG GGG CAG AGA TGA TGA CC	194

## Results

### Generation of transgene-free SCAP iPSCs

**Figure 1:**
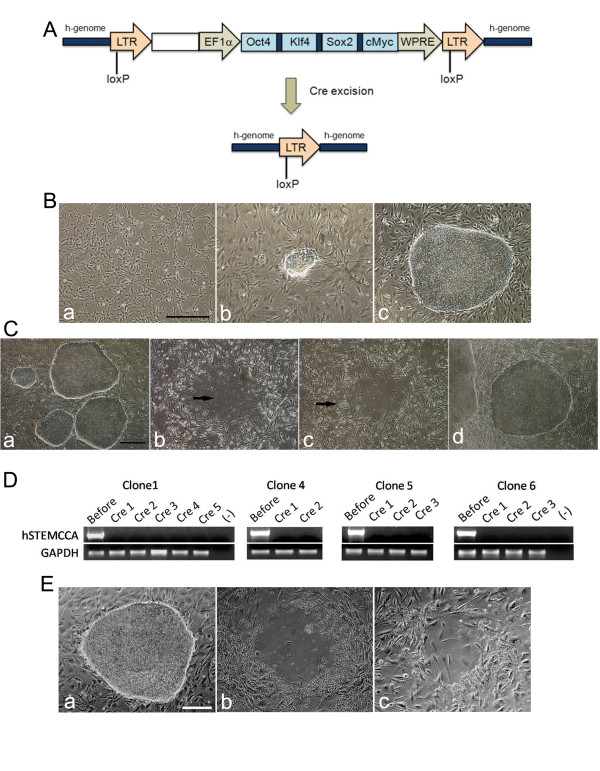
**Generation of transgene-free SCAP iPSCs**. (**A**) Schematic representation of the hSTEMCCA-loxP lentiviral vector integrated into the human cell genome (h-genome) after transduction of SCAP. A loxP site was introduced within the U3 region of the 3' LTR. Following the exposure of pHAGE2-Cre-IRES-PuroR plasmid, the entire cassette was excised. (**B**) Emergence of SCAP iPSCs during reprogramming. (B-a) SCAP at passage (p) 0 before transduction. (B-b) SCAP at p3 were transduced with hSTEMCCA-loxP lentiviral vector and seeded onto MEF. A small ESC-like colony emerged on day nine post transduction. (B-c) A SCAP ESC-like colony on day 28 post transduction on MEFs. The ESC-like colony is considered at p0. (scale bar: 500 μm). (**C**) Excision of hSTEMCCA. (C-a) Established SCAP iPSC colonies at p2 before transgene excision. (C-b) After pHAGE2-Cre-IRES-PuroR plasmid transfection and 48 hours of pluromycin treatment, most SCAP iPSC colonies died out leaving behind an empty space (arrow). (C-c) New colonies (arrow) emerged on day 1 after Cre-excision and puromycin selection. (C-d) Expanded SCAP iPSC colonies passaged twice after Cre-excision. (scale bar: 500 μm). (**D**) Excision of hSTEMCCA was verified by RT-PCR. Clones after cre-excision showed no detectable hSTEMCCA. *GAPDH *served as a control. (**E**) A clone of TF-SCAP iPSCs grown on DR4 MEFs was treated with puromycin (1.2 μg/mL) for up to a week. (Ea) Before the addition of puromycin. (Eb) On day 2 after the addition of puromycin. (Ec) On day 4 after puromycin treatment. (scale bar: 250 μm). ESC, embryonic stem cells; iPSCs, induced pluripotent stem cells; MEF, mouse embryonic fibroblasts; SCAP, stem cells from apical papilla.

Primary human SCAP showing typical fibroblast morphology were transduced with lentiviral vectors hSTEMCCA (Figure 1A) and seeded onto MEFs. Within one to two weeks after transduction, ESC-like colonies emerged on MEFs (Figure 1B). The rate of formation of ESC-like clones was 0.11% ± 0.01 (% number of ESC-like colonies/number of seeded transduced cells) based on four independent experiments using SCAP from two different donors.

Before transfection of Cre-IRES-PuroR plasmids, SCAP iPSCs showed typical hESC morphologies. After transfection and 48 hours of puromycin treatment, most of the colonies underwent cell death. Within two to four days of recovery, newly emerging colonies were observed around the margin of the original colonies, as indicated in Figure 1C. The PCR results demonstrated the deletion of hSTEMCCA vector. In three repeated experiments, using SCAP iPSCs from different clones, 100% deletion of hSTEMCCA vector was found in these newly emerged subclones (Figure 1D).

To determine whether there was integration of pHAGE2-Cre-IRES-PuroR plasmid DNA into the genome of TF-SCAP iPSCs, these cells were grown on DR4 MEFs in the presence of puromycin (1.2 μg/mL) for up to a week. On day two after the addition of puromycin, TF-SCAP iPSCs underwent cell death leaving behind an empty space in the DR4 MEF cultures (Figure 1E). No new iPSC colonies emerged after seven days of observation, indicating that TF-SCAP iPSCs contained no pHAGE2-Cre-IRES-PuroR plasmid DNA in their genome.

### Protein expression of hESC associated gene markers

**Figure 2:**
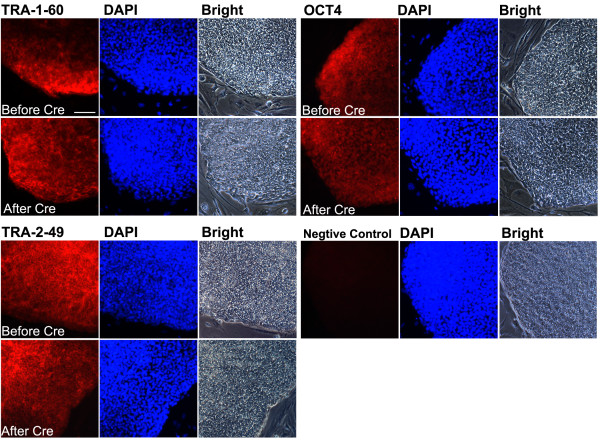
**Expression pluripotency markers by SCAP iPSCs *in vitro***. Embryonic stem cell-associated genes TRA-1-60, TRA-2-49 and OCT4 expressed by SCAP iPSCs before and after Cre-excision. Before Cre: SCAP iPSC clone 1 at passage (p)15. After Cre: SCAP iPSC-Cre clone1 at p12. Expressed genes stained in red; DAPI, nuclear stain. (scale bar: 100 μm for all staining images). DAPI, 4',6-diamidino-2-phenylindole; iPSCs, induced pluripotent stem cells; SCAP, stem cells from apical papilla.

Following Cre-mediated transgene excision, we assessed the expression of hESC-associated genes. Immunocytofluorescence analysis indicated that SCAP iPSCs expressed TRA-1-60, TRA-2-49, and OCT4 before and after excision of hSTEMCCA, as shown in Figure 2.

### Teratoma formation

**Figure 3:**
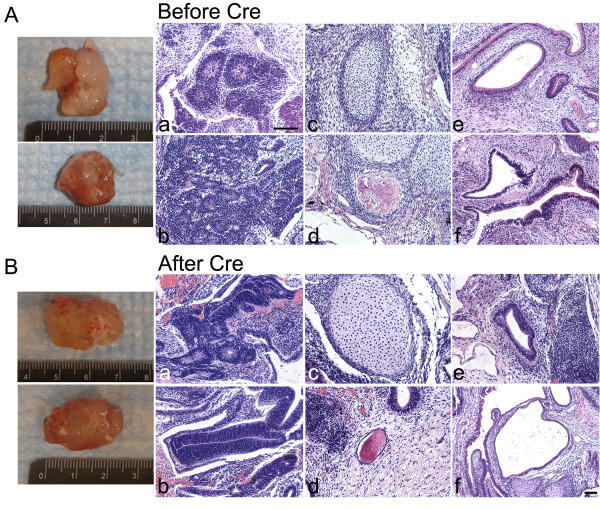
**Histological analysis of teratomas**. Images of removed teratomas from mice are shown on the left of the image panels. (**A**) Multi-differentiated tissues derived from SCAP iPSCs (passage (p)11) before Cre-excision. (**B**) Teratomas from SCAP iPSCs (p10) after Cre-excision. (A & B) (a,b) mainly neural tissues, neural rosettes (ectoderm); (c) mainly cartilage (mesoderm); (d) bone tissue (mesoderm); (e,f) mainly glandular epithelium and endodermal tissues. (scale bar: 100 μm for all histologic images. Except Bf, all histologic images use the scale bar in Ba). iPSCs, induced pluripotent stem cells; SCAP, stem cells from apical papilla.

To test the pluripotency of TF-SCAP iPSCs *in vivo*, teratoma formation analysis was performed. As demonstrated in Figure 3, both SCAP iPSCs before and after hSTEMCCA excision formed teratomas in SCID mice. They developed into primitive tissues representing all three germ layers including neural tissues (ectoderm), cartilage and bone (mesoderm), and glandular or respiratory epithelial-like layers (endoderm). There were numerous ectodermal neuroepithelial-like tissues including pigmented retinal epithelium-like tissues as well as glandular structures. These teratoma histologic images presented in Figure 3 are typical primitive tissues representing the three germ layers. Immunohistochemical staining to target specific germ layer markers is presented in Additional file 1 describing the Materials and Methods and Additional file 2, Figure S1, showing the expression of the ectoderm marker βIII-tubulin, mesoderm marker α-smooth muscle actin (αSMA) and endoderm marker AFP in teratomas formed by our previously generated SHED iPSCs that carried Thomson's four factors [5]. The immune-stained tissues shown in the Supplemental Figure 1 in Additional file 2, especially those that represent endodermal (glandular) and ectodermal (neural) tissues, indicate that the teratoma tissues presented in Figure 3 are typical tissues of the three germ layers.

### *In vitro *neural differentiation

**Figure 4:**
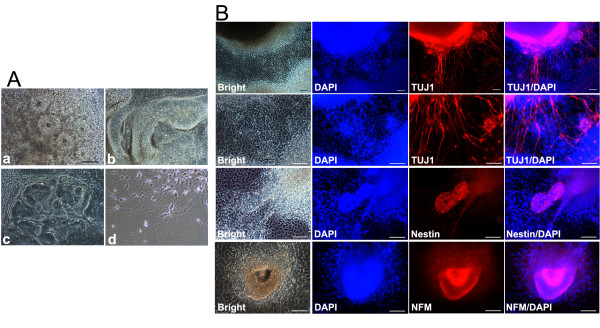
**EB-mediated neural differentiation of TF-SCAP iPSCs *in vitro***. (**A**-a to Ad) Four-day-old EBs were plated into Matrigel-coated wells of six-well plates. On day 8 after neurogenic stimulus, differentiated iPSCs developed into neural rosette morphology. Some cells extended elongated cell cytoplasmic processes resembling axons. (scale bar: 100 μm for all images in A) (**B**) Immunofluorescence analysis of differentiated neural-like cells showing positive staining of neuronal marker βIII-tubulin (TUJ1). Cells forming a neural rosette or spherical morphology were also positive for nestin and NFM. Expressed genes stained in red; DAPI, nuclear stain. (scale bar: 100 μm). DAPI, 4',6-diamidino-2-phenylindole; EB, embryoid body; iPSCs, induced pluripotent stem cells; SCAP, stem cells from apical papilla; TF, transgene free.

The TF-SCAP iPSCs were tested for their neurogenic potential using an EB-mediated protocol. Under the stimulation of a neurogenic medium, cells from the EBs developed into neural rosette morphology. These neural rosettes formed in cultures highly resembling those neural tissues formed in teratomas. Neural tube-like structures consisting of columnar cells aligned as palisades, that also frequently appeared in teratomas (Figure 3B-a,b; Figure 4A-b,c), were observed. At a higher magnification, individual cells with elongated cellular processes similar to axon structures could be identified (Figure 4A-d). To determine whether these cells that morphologically resembled neuronal cells expressed neuronal genes, cells were stained with the neural stem cell marker nestin, early stage neuronal marker βIII-tubulin and mature stage neuronal marker NFM. As shown in Figure 4B, the neural rosettes or some cell aggregates were positive for nestin or NFM, and the elongated cytoplasmic processes were positive for βIII-tubulin, indicating the neurogenic potential of TF-SCAP iPSCs.

**Figure 5:**
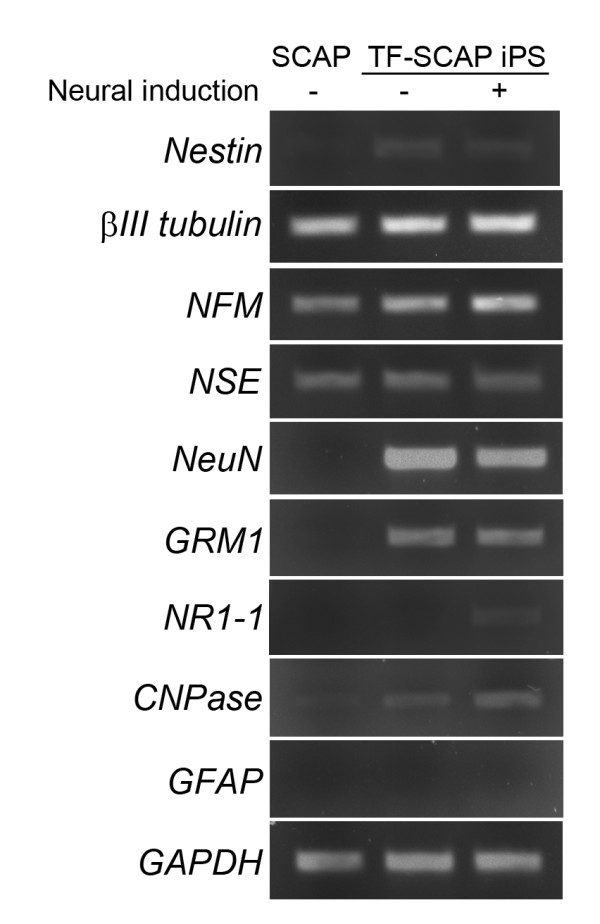
**RT-PCR analysis of the expression of neural markers**. The neurogenic induction followed the same method as that for samples shown in Figure 4. The SCAP are the original cells from which TF-SCAP iPSCs were derived. *GAPDH *was used to ensure equal loading of the samples. (+), presence of neurogenic induction medium; (-), regular culture medium without neurogenic induction. iPSCs, induced pluripotent stem cells; SCAP, stem cells from apical papilla; TF, transgene free.

We determined a number of neural markers with RT-PCR. We first passaged iPSCs into MEF-free Matrigel plates before undergoing EB formation to avoid MEF contamination. We tested the RT-PCR using MEF RNA and found that some human neural gene primers can amplify mouse genes. Using MEF-free cultures eliminated/reduced such possibilities. The results presented in Figure 5 show that several neuron associated genes, *βIII-tubulin, NFM and NSE *were already expressed in SCAP without neurogenic induction. After reprogramming into iPSCs, these genes remain expressed and their levels did not appear to have a noticeable increase. *NeuN *was not detected in SCAP while it dramatically increased after becoming iPSCs with or without neurogenic induction. *Nestin *was barely detectable in SCAP while its expression increased after becoming iPSCs and remained detectable after neurogenic induction. Glutamate receptors glutamate receptor, metabotropic 1 (*GRM1*) and glutamate receptor, ionotropic, N-methyl D-aspartate 1 (*NR1-1*) could not be detected in SCAP but were detectable after becoming iPSCs. *GRM1 *was expressed in iPSCs with or without neurogenic induction and *NR1-1 *was only detectable after induction. The glia cell marker genes *CNPase *and glial fibrillary acidic protein *(GFAP) *are regulated differently in SCAP and SCAP iPSCs. *CNPase*, as the oligodendrocyte marker, was slightly expressed in SCAP and the level became elevated when turning into iPSCs. The expression level appeared to further increase slightly after neurogenic induction. *GFAP*, the astrocyte marker, on the other hand, was not detected in SCAP and SCAP iPSCs (with or without induction).

## Discussion

The present study demonstrated a successful generation of TF-SCAP iPSCs characteristic of typical iPSCs capable of teratoma formation and neurogenic differentiation. Using the polycistronic hSTEMCCA-loxP vector carrying all four reprogramming genes, we were able to consistently reprogram SCAP into iPSCs and remove the transgene/vector with a Cre-mediated approach.

We previously generated iPSCs from dental stem cells including SCAP using either the Thomson's four reprogramming factors (human) with the viral vector system pSin-EF2-gene-Pur each carrying one of the four factors *LIN28, NANOG, OCT4, and SOX2; *or the Yamanaka's four factors (human) *c-MYC, KLF4, OCT4 *and *SOX2 *each into the vector pMXs [5]. While these dental iPSCs demonstrated typical iPSC characteristics including teratoma formation, they have unfavorable features when being guided into certain differentiation pathways. These transgene-carrying iPSCs (pSin-EF2-gene-Pur vector system) underwent massive cell death (data not shown) after differentiation toward MSC lineages using a reported protocol established for deriving MSCs from hESCs [32]. We used the same protocol to guide hESCs (H1 and H9) and were successful in generating MSC-like cells without witnessing any cell death phenomenon. Furthermore, the survival rate of these transgene-carrying dental iPSCs was extremely low after freezing and thawing. Most of the frozen-down dental iPSCs generated using the pSin-EF2-gene-Pur vector system did not survive after thawing. In contrast, TF-SCAP iPSCs were able to recover better after freezing/thawing and they were able to differentiate into MSC lineages without cell death (data not shown). Earlier when we attempted reprogramming dental stem cells using the pLenti6.2/C-Lumio/V5-DEST vector system containing a CMV promoter [5], we also observed cell death phenomenon. As we reported, this vector system never succeeded in generating iPSCs. Instead, it caused some cell death at the very beginning, and those cells that survived turned into iPSC-like cells and then underwent cell death also [6]. It is possible that the hSTEMCCA-loxP vector is a good match for dental stem cell reprogramming.

The reprogramming rate using hSTEMCCA-loxP is higher than our previous attempts using pSin-EF2-gene-Pur vectors. Using hSTEMCCA-loxP, we also reprogrammed human foreskin fibroblasts (ATCC) and human gingival fibroblasts (from one donor) and the reprogramming rates were 0.016% and 0.07%, respectively, based on one experiment. Reprogramming human DPSCs in two independent experiments yielded us reprogramming rates of 0.6% and 0.083%. In our experimental settings either using pSin-EF2-gene-Pur vectors previously or hSTEMCCA-loxP in the present studies, dental stem cells have shown a higher reprogramming rate than fibroblasts. One condition that favors the reprogramming efficiency is the division rate of the cells being reprogrammed [33]. The high proliferation rate of dental stem cells including SCAP and the polycistronic gene cassette hSTEMCCA-loxP vector offer a high reprogramming efficiency over existing multi-vector approaches for skin fibroblasts [33]. It likely derives in part from favorable stoichiometry of the four reprogramming transcription factors when expressed in this particular order by a polycistronic system [15].

Following the generation of SCAP iPSCs *via *hSTEMCCA-loxP, we applied a plasmid expressing Cre to excise the reprogramming transgenes. Deletion of the hSTEMCCA was found in 100% of these clones, indicating that Cre-mediated excision was very effective. Additional transfection of plasmids carrying Cre does not lead to integration of the plasmid into the genome, verified by the observation that puromycin treatment of the TF iPSC clones led to total cell death. It should be noted, however, that an inactive fragment of the viral long terminal repeat (LTR) remains in the host genome after Cre-excision. Although highly unlikely, there may theoretically be a risk of insertional mutagenesis. This could be reduced by targeting the hSTEMCCA into a safe genomic locus [30]. Further investigation would be needed to find approaches which would obtain not only transgene-free and completely vector-free iPSCs, but also maintain a high reprogramming rate in order to utilize iPSCs for clinical applications.

The SCAP iPSCs maintained their pluripotency after Cre-excision as indicated by the *in vitro *expression of ESC-associated gene markers and the teratoma formation which is consistent with previous studies [30]. TF-SCAP iPSCs have been in continuous culture for six months after the initial transduction and maintain a morphology that is similar to hESCs. The iPSCs from different cell types may be different in their abilities to undergo guided differentiation directions [34], and iPSCs were reported to retain a transcriptional memory of their tissue of origin [35]. In addition, in our present study along with our reported work [5], there were numerous neuroepithelial-like tissues in teratomas derived from both TF-SCAP iPSCs and SCAP iPSCs, suggesting that iPSCs from SCAP may have robust potential to differentiate into neural tissues. Our *in vitro *neural differentiation studies indicated that TF-SCAP iPSCs can be guided to form neural-like cells and express neural genes. It is interesting to find that the expression of *βIII-tubulin, NFM, NSE and NeuN *was already detectable at considerable levels at the pluripotent state before neurogenic stimulus and there was no noticeable increase after stimulation (at best, *NFM *had a slight increase). The oligodendrocyte marker *CNPase *was also detected in uninduced SCAP and its expression was increased in SCAP iPSCs and further elevated after induction. Dental stem cells including DPSCs and SCAP express neural markers such as nestin and βIII-tubulin in cultures without neurogenic stimulus [36,37]. It may be that this gene expressing signature is retained after being reprogrammed into iPSCs; however, it will be a major undertaking to verify such a possibility. Although the two glutamate receptors *GRM1 *and *NR1-1 *that function at synapses to transmit neural signals were not expressed in SCAP, they can be detected after being reprogrammed into iPSCs or after neurogenic induction. Taken together, our establishment of TF-SCAP iPSCs and the initial confirmation of their neurogenic potential using a simple neurogenic induction protocol warrant further exploration of their abilities in neurogenesis as well as neural tissue regeneration *in vivo*.

## Conclusions

TF-iPSCs SCAP can be effectively and consistently generated using the hSTEMCCA-loxP vector system followed by Cre-mediated excision. These TF iPSCs maintain ESC characteristics and can differentiate into neural-like cells expressing neural marker genes *in vitro*. Further investigation will test whether they are a good cell source for *in vivo *neural tissue regeneration.

## Abbreviations

bFGF: basic fibroblast growth factor; BSA: bovine serum albumin; CFU-F: colony formation units of fibroblastic cells; CNPase: 2', 3'-cyclic nucleotide-3'-phosphodiesterase; (D)MEM: (Dulbecco's) modified Eagle's medium; DPSCs: dental pulp stem cells; EBs: embryoid bodies; ESCs: embryonic stem cells; FBS: fetal bovine serum; GAD: glutamic acid decarboxylase; GFAP: glial fibrillary acidic protein; GRM1: glutamate receptor: metabotropic 1; H & E: hematoxylin and eosin; hESC: human embryonic stem cell; hSTEMCCA-loxP: (human) a single lentiviral 'stem cell cassette' flanked by loxP site; iPSCs: induced pluripotent stem cells; LTR: long terminal repeat; MEFs: mouse embryonic fibroblasts; MSC: mesenchymal stem cell; NeuN: neuronal nuclei; NFM: neurofilament medium chain; NR1-1: glutamate receptor, ionotropic, N-methyl D-aspartate 1; NSE: neuron-specific enolase; RT-PCR: reverse transcriptase - polymerase chain reaction; SCAP: stem cells of apical papilla; SHED: stem cells from human exfoliated deciduous teeth; TF-iPSCs: transgene-free iPSCs; TU: transducing unit.

## Competing interests

The authors declare that they have no competing interests.

## Authors' contributions

XYZ designed and performed the experimental work, acquired and assembled the data, and prepared the manuscript. HYY, ZY, XY and XBT performed the experimental work and assembled data. GTJH conceived, designed and supervised the overall project, analyzed and interpreted the data and finalized the manuscript. All authors have read and approved the manuscript for publication.

## Supplementary Material

Additional file 1 **Supplemental document: supplemental M&M figure legend**. Click here for file

Additional file 2 **Figure S1**. Supplemental figure: Supplemental figure flat rev. Click here for file
