# Supplementary material for: Establishment of transgene-free induced pluripotent stem cells reprogrammed from human stem cells of apical papilla for neural differentiation
Source: Stem Cell Res Ther. 2012 Oct 24;3(5):43. doi: 10.1186/scrt134 (PMC3580434; doi:10.1186/scrt134)
Supplement: Additional file 1 — Supplemental document: supplemental M&M figure legend. [file scrt134-S1.DOCX]

**Establishment of Transgene-Free iPS Cells**

**Reprogrammed from Human SCAP for Neural Differentiation**

Xiao-Ying ZOU, Hsiao-Ying YANG, Zongdong YU, Xiao-Bing TAN, Xing YAN and

George T.-J. HUANG

**Supplemental Materials and Methods**

Immunohistochemistry

Paraffin embedded teratoma tissue sections were baked at 58°C for 1 h and deparaffinized in xylene followed by a standard rehydration process in alcohol/water mixtures with ascending concentrations of water (100%, 95%, 70% and 50% alcohol). After rehydration, the sections were incubated in 3% H_2_O_2_ solution in methanol at room temperature for 10 min to block the endogenous peroxidase activity. The sections were then rinsed with PBS and undergone antigen retrieval in 500 mL of 10 mM citrate buffer, pH 6.0 in a glass jar which was placed in a microwave oven and heated for 4 min (visible boiling of the buffer). After which the samples in the jar were allowed to cool at room temperature for 20 min followed by twice of 300 ml PBS washes, 5 min each. Each section was then incubated in a blocking bluffer (Normal Horse Serum, VECTASTAIN Elite ABC Kit – Universal; Vector laboratories, Burlingame, CA) and in a humidified chamber at room temperature for 1 h. The blocking buffer was then drained off and the sections incubated in primary antibody (in antibody dilution buffer, 0.5% serum albumin in PBS; antibody dilution based on manufacturer instructions) in a humidified chamber at room temperature for 1 h or 4°C overnight. The primary antibodies used were against the markers representing three germ layers: Rabbit anti-human α 1 fetoprotein (AFP) antibodies (endoderm), rabbit anti-human α smooth muscle actin (αSMA) antibodies (mesoderm) (both antibodies from Abcam Inc., Cambridge, MA), and mouse anti-human βIII-tubulin antibodies (ectoderm) (Sigma-Aldrich, St. Louis, MO). The sections were then washed and incubated in prediluted universal biotinylated anti-mouse/rabbit IgG secondary antibody (Vector Laboratories) for 1 h at room temperature. After washing, avidin-peroxidase-complex was added and incubated for 30 minutes followed by washing and the addition of peroxidase substrate solution for 5 min according to manufacturer instructions. Sections were counterstained with Mayer’s hematoxylin solution (Sigma-Aldrich, St Louis, MO), washed with water and mounted in aqueous mounting medium (Electron Microscopy Service, Hatfield, PA). For the negative control sections, the non-immune antibodies were used.

**Supplemental Figure Legends**

**Supplemental Fig. 1.** Immunohistochemical staining of markers representing three germ layers. SHED iPSCs carrying Thomson’s four factors were transplanted into the SCID mice to form teratomas. The resected teratomas were processed and embedded in paraffin blocks which were then sectioned for immunohistochemical staining (brown stain). Images showing detection of βIII-tubulin representing ectoderm (A, B); αSMA representing mesoderm (C,D); and AFP representing endoderm (E,F). Scale bars: (A.C) 100 µm; (D,E) 200 µm; (B.F) 50 µm.
