# Supplementary figures and images for: Establishment of transgene-free induced pluripotent stem cells reprogrammed from human stem cells of apical papilla for neural differentiation
Source: Stem Cell Res Ther. 2012 Oct 24;3(5):43. doi: 10.1186/scrt134 (PMC3580434; doi:10.1186/scrt134)

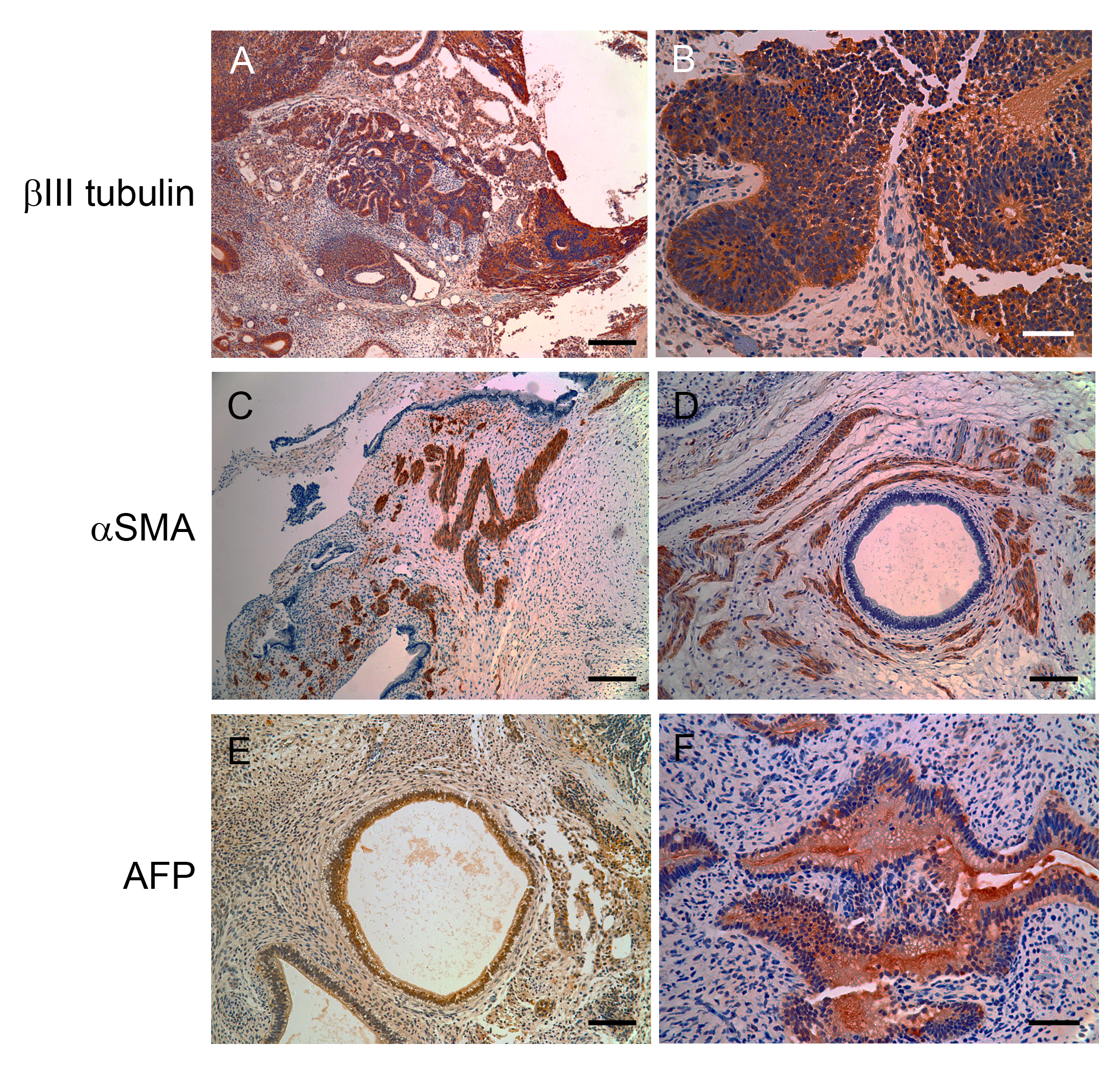

Supplement: Additional file 2 — Figure S1. Supplemental figure: Supplemental figure flat rev. [file scrt134-S2.TIFF]
